# The Colour, Composition and Eating Quality of Beef from Late- or Early-Maturing Suckler Bulls Finished at Pasture with or without Concentrate Supplementation

**DOI:** 10.3390/ani12182417

**Published:** 2022-09-14

**Authors:** Aidan P. Moloney, Shannon S. Wilson, Sibhekiso Siphambili, Lara Moran, Edward G. O’Riordan, Maurice G. O’Sullivan, Joseph P. Kerry, Frank J. Monahan, Mark McGee

**Affiliations:** 1 Animal & Grassland Research and Innovation Centre, Teagasc, Grange, Dunsany Co., C15 PW95 Meath, Ireland; 2 School of Food and Nutritional Sciences, University College Cork, T12 K8AF Cork, Ireland; 3 School of Agriculture and Food Science, University College Dublin, D04 V1W8 Dublin 4, Ireland; 4 Teagasc Food Research Centre, Ashtown, D15 DY05 Dublin 15, Ireland; 5 Lactiker Research Group, Department of Pharmacy and Food Science, University of the Basque Country (UPV/EHU), 01006 Vitoria-Gasteiz, Spain

**Keywords:** bulls, grazing, colour, composition, eating quality

## Abstract

**Simple Summary:**

Consumers of beef are increasingly interested in how it is produced. There is a perception that grass-based systems of production have enhanced animal welfare and are more environmentally friendly than conventional systems. This has resulted in the development of “grass-fed” labelled beef which captures a premium in the marketplace. Bulls have traditionally been considered unsuitable for grass-based production systems, but they have growth advantages when compared with steers. However, eating quality or palatability, is a major influence on the decision of the consumer to continue to purchase beef, as is the colour of beef in the display case. There is a view in the meat industry that bull carcasses must achieve a threshold level of fatness for beef to be acceptable to the consumer. This is a challenge if bulls are of late-maturing breed types, but early maturing breed types might be more suited to a grass-based production system. Both breed types were compared and whether supplementary concentrates are required to meet the industry fatness specification. The findings indicate that achieving this specification was not required to ensure acceptable eating quality and that either breed type can be used, without supplementation, to access the lucrative “grass-fed” beef market.

**Abstract:**

Carcasses from pasture-finished early-maturing (EM), rather than late-maturing (LM), breed bulls may be more suited to meet the minimum carcass fatness classification of 2+ (6.0 on a 15-point scale) required for some markets. The comparative colour and eating quality of beef from grass-fed bulls of different maturities are unknown. Sixty yearling suckler-bred bulls were assigned to a 2 (maturities: EM and LM) × 2 (finishing strategies: grass only (G0) or grass + 4.0 kg concentrate daily (GC)) factorial design. Bulls were at pasture from 7 April, concentrates were introduced (or not) 97 days later, and bulls were slaughtered at 192 d post-turnout (approximately 19 mo of age). Carcass fat scores averaged 5.02, 6.20, 6.33 and 7.30 for LMG0, LMGC, EMG0 and EMGC bulls, respectively. Muscle colour did not differ between treatments. Muscle from LM had lower intramuscular fat concentration, collagen solubility and a tendency (*p* < 0.1) towards lower ratings for tenderness, texture, and acceptability of 14 d aged beef. Concentrate supplementation decreased the ratings for muscle tenderness but ratings for acceptability were not affected. Achieving the minimum carcass fatness was therefore not required to produce beef of acceptable eating quality and suckler bulls can access the “grass-fed” beef market.

## 1. Introduction

Beef from pasture-based production systems is growing in popularity. Contributory factors include a perception that the cattle are managed in a more environment and animal welfare-friendly system [1] and that beef from pasture-based, rather than intensive production, systems aligns more closely with human nutrition guidelines with respect to fat consumption [2]. “Grass-fed” and “grass-based” are among the labels used to capture this interest of beef consumers. In countries with a temperate climate that facilitates the production of high yields of high nutritive value pasture that cattle can consume over a relatively long grazing season, there is an opportunity to access this premium market. 

In Ireland, steer production predominates but their growth and feed efficiency advantages make bull beef production systems attractive alternatives for producers of steer beef [3]. Some markets accept bulls from the suckler beef cow herd if the animals are less than 20 months of age at slaughter and have a carcass fat classification of ≥2+ according to the EU Beef Carcass Classification Scheme (EUROP) [4]. This is equivalent to ≥ 6 on a scale where 1 is the leanest and 15 is the fattest. This carcass fatness specification appears to be based on a perception that meat from bulls with a lower fatness classification *per se* is inferior in some quality characteristics, and since carcasses from grass-fed bulls tend not to achieve the required fat classification, the production of bulls from pasture is not encouraged. However, Moran et al. [5] and Moloney et al. [6] reported that within a 16 mo and 20 mo late-maturing (LM) breed suckler bull production system, respectively, meat from grass-fed bulls that did not achieve the desired carcass fat classification had similar sensory characteristics to meat from concentrate-finished bulls that exceeded the required fatness specification. In the latter study, supplementing grazing bulls with concentrates during the final 100 d of the grazing season resulted in a non-significant increase in average carcass fat score (from 4.8 to 5.5) and elevated the proportion of carcasses that achieved the fatness specification from 0.21 to 0.55. An option available to the beef producer to maintain the performance advantage of bulls, maximise grass consumption and increase the likelihood of meeting the current carcass fat specification is to use an early maturing (EM), rather than a LM, breed type. 

Regan [7] reported that concentrate supplementation increased the mean carcass fat score of grazing, LM suckler bulls (from 5.0 to 6.2), whereas the mean fat score of unsupplemented EM grazing bulls (6.33) met the required specification. There is a perception that beef from the progeny of EM sires is of superior eating quality and striploins are often “breed”-branded and attract a premium price [8]. Meat colour is also an important influence on the purchasing decision of the consumer [9]. Mezgebo et al. [10] compared beef from ‘adequately finished’ EM and LM breed sired suckler bulls, that received a high-concentrate diet following 100 d at pasture, and found no difference in colour and although tenderness and juiciness were higher for EM, the differences were small and overall acceptability was similar. 

As there is a paucity of data on the relative sensory characteristics and colour of beef from LM and EM suckler bulls that were at pasture until slaughter, with or without concentrate supplementation, the objective of this study was to determine those characteristics in the animals produced by Regan et al. [7] and to explore possible mechanisms for any differences observed.

## 2. Materials and Methods

### 2.1. Animal Management and Sampling Procedures

This study was licensed by the Irish Government Department of Health and Children (B100/2483), approved by the Teagasc Animal Ethics Committee (Teagasc Animal Ethics Committee, approval #78/2014) and all procedures complied with national and EU regulations concerning experimentation on farm animals. 

The animals and their management are described in detail in Regan [7]. In brief, thirty spring-born EM and thirty spring-born LM breed sired-bulls, approximately 8 months of age, were sourced from commercial beef suckler herds in October. Following arrival at Grange Research Centre the animals were weighed, treated for the control of internal and external parasites, and vaccinated against Clostridial and respiratory diseases. During the indoor winter period, animals were offered grass silage *ad libitum*, and supplemented with 1.6 kg dry matter (DM) daily of one of two concentrate types. At the end of the winter, the bulls were weighed on two consecutive days and blocked by breed, previous winter supplement type and weight, and assigned at random within block to a two (breed maturity; EM or LM) by two (concentrate supplementation; none (G0) or 3.2 kg DM at pasture (GC)) factorial arrangement of treatments (*n* = 15/treatment). After turnout to pasture, the animals rotationally grazed separate *Lolium perenne-*dominant paddocks for 192 d. Concentrates (862 g rolled barley, 60 g soya bean meal, 50 g sugar cane molasses and 28 g of a mineral and vitamin mixture per kg) were offered to the appropriate groups, beginning at 97 d after turnout. Each paddock area was adjusted such that residency time and, pre- and post-grazing sward heights were similar for all treatment groups. 

### 2.2. Slaughter, Sampling Procedures, pH and Colour Measurement

Animals were weighed on two consecutive days prior to slaughter (including the morning of slaughter). Bulls were slaughtered at a mean age of 19.3 mo. To facilitate post mortem measurement and sample collection, bulls were slaughtered on 3 consecutive weeks, balanced for treatment and block. On the day of slaughter, the animals were transported, without mixing of treatment groups, to a commercial abattoir (25 km) and slaughtered within 1 h of arrival by bolt stunning followed by exsanguination from the jugular vein. Electrical stimulation was not applied, and carcasses were hung by the Achilles tendon. Post-slaughter, carcasses were weighed and graded for conformation (15-point scale, classes E+ (highest) to P− (lowest), E+ is 15) and fatness (15-point scale, scores 5+ (highest) to 1− (lowest), 5+ is 15) according to the EU Beef Carcass Classification Scheme [4]. Within 1 h of slaughter, carcasses were placed in a chill set at an average, across the three slaughter events, of 4.3 °C (s.d. 0.58) and the ambient temperature close to the carcasses was monitored (average 3.6 °C, s.d. 0.82). The pH and temperature decline of the *longissimus thoracis* muscle (LT) at the 10th rib were recorded in the left side carcass [11]. After approximately 5.8 h (s.d. 1.08), the chill temperature was decreased to 0 °C. At 48 h post mortem, the ribs joint (ribs 6 to 10) was removed from the right side of each carcass, from which the LT was collected, vacuum-packaged, transported to Teagasc, Food Research Centre, (Ashtown, Dublin), and stored at 2 °C. After a further 24 h, the ultimate pH was measured, and the LT cut into individual steaks (thickness 25 mm). The first steak was used for colour determination. Thus, steaks were wrapped in oxygen-permeable polyvinylchloride film (oxygen permeability of 580 mL/m^2^ h at standard temperature and pressure) and allowed to bloom at 4 °C for 1 and 24 h. CIE L* (lightness), a* (redness) and b* (yellowness) were measured through the film at three locations on each muscle, using a dual beam spectrometer (UltraScan XE; Hunter Laboratories, Reston, VA, USA) and averaged. Hue angle (H*) and Chroma (C*) values were also recorded. The remaining steaks were vacuum-packed after cutting. Samples for chemical composition determination were stored at −20 °C. Samples for Warner–Bratzler shear force (WBSF) variables, cook loss and sensory analysis, were wet-aged for 11 additional d (2 °C) (total of 14 d ageing) and then frozen at −20 °C.

### 2.3. Meat Chemical Analysis, Instrumental Texture and Sensory Evaluation

The WBSF was measured as previously described [12]. For compositional analysis, samples were first homogenised (R301 Ultra, Robot Coupe SA, Vincennes, France). The proximate composition was measured as described by Moran et al. [11]. Total and heat-soluble (77 °C, 65 min) collagen concentrations were measured as hydroxyproline as described by Kolar [13]. For proteoglycan analysis, 30 g of homogenised muscle were lyophilised (Edwards Supermodulyo F056, pressure 0.02 atm, condenser temperature −60 °C, Fisher Scientific, Dublin, Ireland). The glycosaminoglycan (GAG) content was measured according to [14] with some modifications. Thus, the lyophilised muscle was converted to a powder using a coffee grinder (Model E5602SS, Lloytron, PLC, Lancashire, UK) and 100 mg were incubated overnight at 56 °C with 1 mL extraction buffer (50 mM Tris-HCl, 10 mM NaCl, 3 mM MgCl_2_, pH 7.9) and proteinase K (at a final concentration of 50 µg/mL). The preparation was heated for 30 min at 90 °C to deactivate proteinase K. Deoxyribonuclease (DNase 1) (100 µL in deionised water to give a final concentration of 30 units/mL) was added to the proteinase K digested samples which were incubated for 4 h at 37 ºC. The samples were centrifuged (13,000× *g*, 20 min) and the pellets discarded. Supernatants (100 µL) were mixed with 1 mL of 1,9 -dimethyl-methylene blue (DMMB) dye solution (1.85mM DMMB, 0.2 M guanadine HCl, 0.2% sodium formate, 0.2 % formic acid) and vigorously agitated (Stuart vortex mixer SA8) for 30 min to promote complete complexation of the GAG with DMMB. Thereafter, the mixture was centrifuged (13,000× *g*, 10 min) to separate the insoluble GAG–DMMB complex from the soluble materials, including excess DMMB. The supernatant was removed using a 1 mL disposable pipette. The pellet was redissolved in 1 mL of decomplexation solution (50 mM sodium acetate, 10% propan-1-ol, 4 M guanadine HCl) and the mixture was vigorously agitated for 30 min at room temperature. Absorbance was measured at 656 nm (UVMini-1240 Spectrophotometer, Shimadzu, Japan). The concentration of sulphated GAG was determined using a calibration curve prepared from 100 µL of 0-5 µg/mL chondroitin sulphate solutions mixed with 1 mL DMMB and treated as described for complexation and de-complexation.

### 2.4. Perimysium Connective Tissue Isolation

Intramuscular perimysium connective (PC) tissue was isolated by homogenising 100 g of blended muscle in 5 volumes (*w/v*) of deionized water (4 °C) for 10 s at low speed and 10 s at high speed in a blender with 2 speed settings (Cookworks, Argos, Dublin, Ireland). The homogenate was filtered through a metal sieve (pore size 1 mm^2^) and the material retained on the sieve was deemed the PC. The PC was homogenised three more times in 500 mL deionized water and filtered after each homogenisation. Visible blood vessels were removed by tweezers and PC was blotted dry with Whatman No. 4 filter paper (Fisher Scientific, UK), frozen at −70 °C and lyophilized. The lyophilized PC was stored at −20 °C and protected from light.

### 2.5. Pyridinoline Quantification

The quantification of pyridinoline in PC was carried out according to Bank et al. [15] with minor modifications. Thus, 0.20 g (±0.002 g) of PC were hydrolysed for 22 h at 110 °C (Stuart block heater SBH130D, Cole-Parmer, Stone, Staffordshire, UK) with 8 mL of 6 M hydrochloric acid in a screw cap glass tube flushed with nitrogen gas. An aliquot of the hydrolysate was taken for collagen estimation (above) and an aliquot (2 mL) of the hydrolysate was transferred to an Eppendorf tube and dried under nitrogen for 12 h at 40 °C. The residue was dissolved in 1 mL of deionized water. The samples were centrifuged at 13,600 g (Hettich^®^ Mikro 200R centrifuge, Andreas Hettich GmbH, Tuttlingen, Germany) for 20 min before an aliquot of 200 µL of the redissolved sample was taken and mixed with 400 µL of 0.16% heptafluorobutyric acid in 22% methanol. Pyridinoline was measured using high performance liquid chromatography [16]. Separation of pyridinoline was performed at 25 °C using a C18 column (Agilent Eclipse, XDB-C18, 5 μm, 4.6 × 150 mm, Agilent, Santa Clara, CA, USA) fitted with a corresponding guard column (Agilent). The column was eluted using a binary solvent system where solvent A was 0.16% heptafluorobutyric acid in 22% methanol and solvent B was 0.10% heptafluorobutyric acid in 75% acetonitrile. The elution conditions (1.0 mL/min) were: 0–10 min, 100% solvent A (washing); 10–12 min, 70% solvent A and 30% solvent B (elution of pyridoxine, pyridoxamine standards); 12–14 min, 64% solvent A and 36% solvent B (elution of pyridinoline), and 14–19 min, 100% solvent B (equilibration). Fluorescence of the eluted peaks was monitored at λ_excitation_ = 296 nm and λ_emission_ = 400 nm (Agilent 1260 Infinity fluorescence detector) and data were acquired electronically. The pyridinoline peak was quantified relative to a calibration curve prepared with a commercial pyridinoline standard (Microvue PYD/DPD HPLC Calibrator, Quidel^®^, Pathway Diagnostics, United Kingdom). The molar content of pyridinoline in PC was calculated using a molecular weight of 429 and expressed as mol/mol of PC collagen and as mmol/kg muscle.

### 2.6. Ehrlich Chromogen Crosslink Concentration

The quantification of Ehrlich chromogen in PC was carried out as previously described [17] and expressed as mol/mol of PC collagen and as mmol/kg muscle.

### 2.7. Sensory Assessment

Sensory testing [18] was conducted using naïve assessors (*n* = 12) who ranged in age from 20–50 and who consumed beef regularly. Steaks were grilled to an internal temperature of 72 °C, assigned three-digit random codes and served under standard lighting (LUX, 1000) to assessors as 1cm^2^ pieces, in randomised order [19]. Each assessor was asked to rate the sensory qualities of steak from each animal according to the methodology of the American Meat Science Association [20]. The assessors rated five sensory qualities on a scale (8-point hedonic) from 1-8 for tenderness (3-5 chews) where 1 = extremely tough and 8 = extremely tender, overall flavour where 1 = very poor and 8 = extremely good, overall firmness where 1 = extremely mushy and 8 = extremely firm, overall texture where 1 = very poor and 8 = extremely good and overall acceptability where 1 = not acceptable and 8 = extremely acceptable. Distilled water and unsalted soda crackers were provided to purge the palate of residual flavour notes between samples. 

### 2.8. Statistical Analysis

Data were subjected to analysis of variance using Genstat (16^th^ edition) with a model that had block, breed maturity and concentrate supplementation as main effects and the breed maturity by concentrate supplementation interaction. Muscle colour data were analysed according to a split-plot design with the above effects in the main plot, treatment and block in the main plot and time and time-related interactions in the sub-plot. Sensory data were analysed using the REML procedure of Genstat (16th edition). Block, breed maturity, concentrate supplementation and breed maturity by concentrate supplementation were considered fixed effects and “assessor.breed maturity.concentrate” supplementation a random effect. Multiple analysis of variance using SAS was used to calculate partial correlation coefficients (*p*), from the error sum of squares and cross products (SSCP) matrix, between selected carcass and muscle characteristics and sensory variables of beef.

## 3. Results

### 3.1. Animal Growth and Carcass Characteristics

Mean carcass weight as reported by Regan [7] was 386, 415, 341 and 368 kg for LMG0, LMGC, EMG0 and EMGC, respectively. The corresponding carcass fat scores were 5.02, 6.20, 6.33 and 7.30. The estimated daily grass DM intake per animal for the first 97 days was 7.6 and 6.5 kg for LM and EM, respectively [7]. For the remainder of the grazing period, estimated daily grass DM intake per animal was 10.0 and 8.2 kg for LMG0 and LMGC, respectively, and 8.7 and 7.2 kg for EMG0 and EMGC, respectively. 

### 3.2. Post-Mortem pH and Temperature

**Figure 1:**
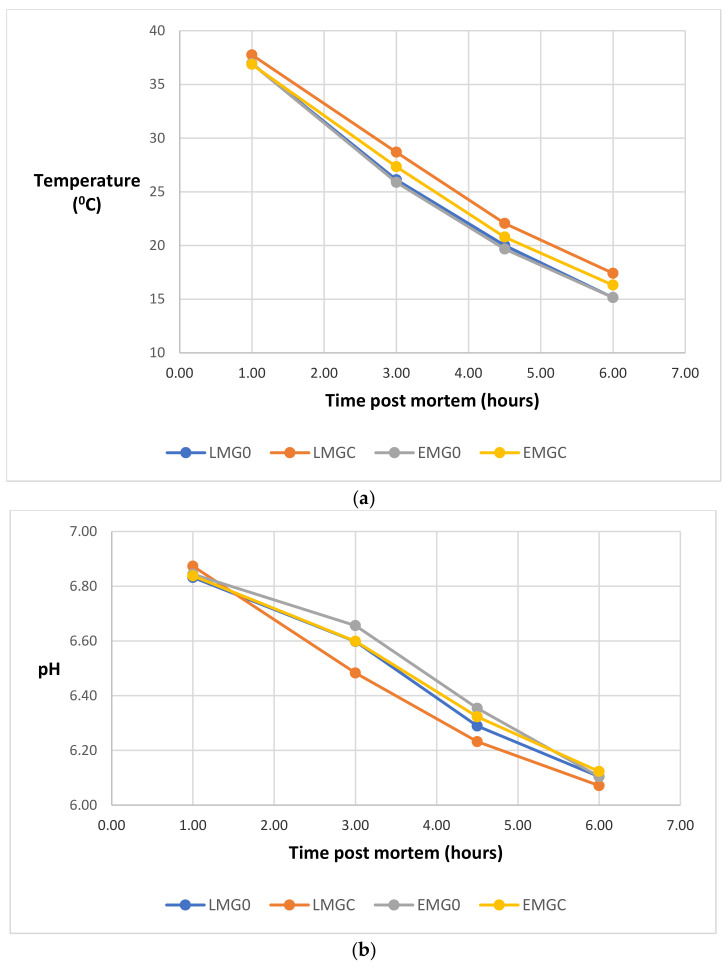
Temperature (**a**) and pH (**b**) decline post mortem in *longissimus thoracis* from late (LM) or early (EM) maturing suckler bulls finished at pasture with (GC) or without (G0) concentrate supplementation. For temperature, s.e.d. at 1, 3, 4.5 and 6 h post mortem was 0.291, 0.542, 0.607 and 0.601, respectively. The corresponding s.e.d for pH was 0.037, 0.061, 0.069 and 0.070.

All stated differences in this and subsequent sections were significant (*p* < 0.05). The pattern of decline in LT temperature post mortem is shown in Figure 1a. There was an interaction at 1 h post mortem such that LT temperature was lower for LMG0 compared to LMGC but there was no difference within the EM groups. Muscle temperature was higher for LM at 1, 3 and 4.5 (*p* < 0.10) hours post mortem compared to EM and at 3, 4.5, and 6 h post mortem for the supplemented compared to the unsupplemented groups. The pattern of decline in LT pH post mortem is shown in Figure 1b. Post-mortem pH at 3 h was lower for LM compared to EM (6.54 vs. 6.63) and for the supplemented compared to the unsupplemented group (6.54 vs. 6.63) but there was no difference between treatments at the other post mortem measurement times or in ultimate pH (72 h) (Table 2).

### 3.3. Muscle Colour

There was no effect of breed maturity or concentrate supplementation on mean lightness, yellowness or chroma of LT averaged over 1 and 24 h of exposure to air and no time-related interaction for these variables (Table 1). On average, LT exposed to air for 24 h was lighter, more yellow and had higher chroma than LT exposed to air for 1 h. There was a tendency (*p* = 0.078) however, for an interaction between breed maturity and time of exposure for LT redness such that the longer duration of exposure increased redness to a greater extent for LT from LM compared to EM. There was a tendency (*p* = 0.056) for an interaction between breed maturity and time of exposure for LT hue such that the longer duration of exposure increased hue to a greater extent for LT from EM compared to LM.

### 3.4. Muscle Composition

There were no interactions between breed type and concentrate supplementation for the composition of muscle as measured (Table 2). With respect to breed type, muscle from LM had lower intramuscular fat (IMF) concentration than muscle from EM but moisture and protein concentration did not differ. The total collagen concentration did not differ, but collagen solubility was greater for LT from EM. The proteoglycan concentration or as a proportion of total collagen in LT did not differ. The perimysium collagen concentration was higher in LT from EM. There was no effect on the molar proportions of pyridinoline cross-links or Ehrlich chromogen in the perimysium collagen fraction, consequently, the concentration of both was higher in LT from EM.

Concentrate supplementation increased the concentration of perimysium collagen, pyridinoline cross-links and Ehrlich chromogen in LT but did not affect the other variables measured.

### 3.5. WBSF and Sensory Characteristics

Concentrate supplementation increased WBSF but there was no difference between breed maturities and there was no difference between treatments for cook loss from LT (Table 3). The sensory characteristics of tenderness, texture and acceptability tended (*p* < 0.08) to be rated lower for LT from LM compared to EM but flavour and firmness were each rated as similar (Table 3). Concentrate supplementation decreased the tenderness score and increased (*p* = 0.053) the firmness score but did not affect the scores for the other sensory characteristics measured.

### 3.6. Correlation Analysis

The “simple” correlation between sensory tenderness of LT and WBSF was -0.62, (*p* < 0.001). When adjusted for treatment (breed type and concentrate supplementation) effects, the correlation decreased to -0.58 (*p* < 0.0001). When adjusted for treatment effects, but there were no significant relationships between collagen and sensory characteristics of LT (Table 4).

## 4. Discussion

### 4.1. Context

Currently in Ireland, carcasses from LM breed suckler bulls finished at grass without concentrate supplementation, while complying with one definition of “grass-fed” (220 +/- 40 d, [21]), are more likely to incur a financial penalty compared to EM breed bulls as they are at greater risk of not achieving the required fat classification [6,7]. This specification is based at least in part on the perception that beef from carcasses with a lower fat classification is of inferior eating quality. However, Bonny et al. [22] concluded that there was no significant relationship between EUROP fat classification and the eating quality of striploin (LT muscle) assessed according to the Meat Standards Australia protocol by untrained consumers, within a database of 455 cattle of diverse types. Similarly, Judge et al. [23] found no significant correlation between EUROP fat classification and the eating quality of striploin using trained assessors within a database of 1318 young bulls produced in Ireland. In addition, we have previously shown that compared to grass-fed bulls that did not achieve the desired carcass fat classification, beef sensory characteristics were similar to beef from concentrate-finished bulls that exceeded the required fatness specification, also implying that carcass fat score is a poor indicator of the eating quality of grass-fed bull beef [5,6]. In the absence of a change in this carcass specification, concentrate supplementation and/or the use of EM breeds is the most logical strategy to employ to ensure sufficient subcutaneous fat accretion [7]. However, concentrate supplementation may decrease the attractiveness of beef from this production system to the consumer who prefers solely ‘grass-fed’ beef for environmental or perceived superior nutritional value reasons. If, however, beef eating quality is unaffected, it may not influence particular consumers from a gustatory perspective. The use of EM breeds would also allow the producer to access the premium market where beef is ‘breed-branded’ as “Angus” or “Hereford”. The premium associated with the EM breeds is based on a perception of superior eating quality of steers and heifers but there is a paucity of data on the eating quality of beef from bulls of these breed types, and in particular when produced within a grass-based production system. The primary objective of this study was to determine those characteristics that influence the purchase decision of the beef consumer i.e., colour and eating quality, of the bull beef produced by Regan [7]. The secondary objective was to explore some possible mechanisms for any differences observed in those characteristics.

### 4.2. Meat pH and Colour

**Figure 2:**
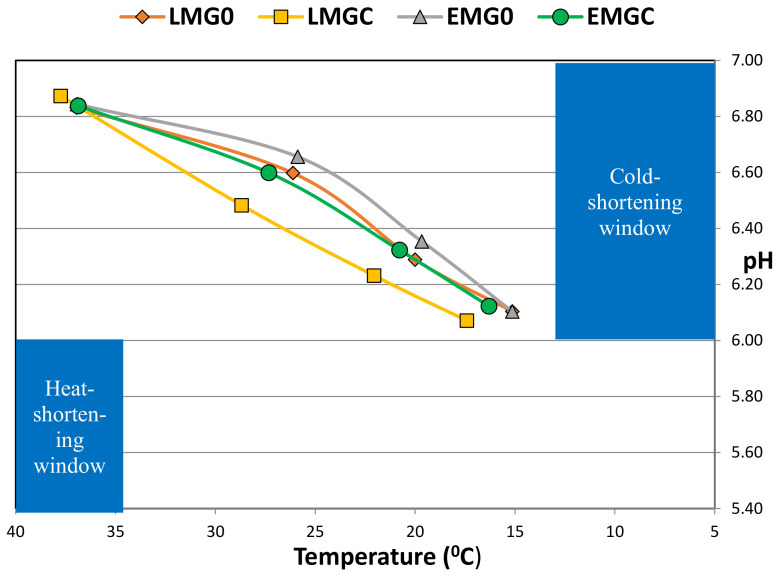
The post mortem pH/temperature “window” in *longissimus thoracis* from late (LM) or early (EM) maturing suckler bulls finished at pasture with (GC) or without (G0) concentrate supplementation (Rab).

The post mortem decline in LT temperature generally reflected the slower cooling in the heavier, fatter carcasses. The post mortem pH profile is mainly determined by muscle glycogen content at slaughter, which in turn is influenced by pre-slaughter nutrition and the stress levels of the animal before and at slaughter [24]. In the present study, the animals were transported to the abattoir in their farm groupings to avoid aggressive behaviour that could occur due to mixing unfamiliar animals and they were slaughtered immediately upon arrival without mixing in the lairage. The minor difference in the post mortem pH profile and the lack of difference in ultimate pH indicates that pre-slaughter stress was minimised. Moreover, LT ultimate pH values from all groups were within the ‘normal’ pH range of 5.4–5.8 [25] and no carcasses were deemed “dark cutters” by abattoir personnel. The post mortem pH-temperature “window’ is considered to be an especially important determinant of tenderness [26]. This window is shown in Figure 2 and it can be seen that on average, carcasses were unlikely to have been affected by “cold-shortening” (muscle pH > 6 at muscle temperature <12 °C), or ‘heat shortening’ (muscle pH < 6 at muscle temperature >35 °C). However, the profile is close to the “cold-shortening” criteria which reflects the chill temperature used in the commercial abattoir and which could increase the risk of toughness in lighter carcasses.

The colour of meat on retail display has a critical influence on consumer purchasing decisions; consumers presume bright red meat to be fresher and of higher quality, whereas pale, discoloured, or darker meat is perceived to be nearing spoilage or of poorer quality [9]. Grass-fed beef is frequently observed to be darker than concentrate-fed beef [27] but the latter generally relates to confined animals fed high-concentrate diets where the opportunity for activity is less than that for their grazing counterparts. The difference in the grazing area allotted to the unsupplemented and supplemented groups in the present study was not sufficient to cause a change in muscle colour. Similarly, Acciaro et al. [28] found no difference in colour of LT from Sarda bulls finished at pasture or indoors on concentrates. The increase in all colour variables with an increase in the duration of aerobic display is consistent with Moran et al. [29]. The tendency towards an interaction between breed maturity and duration of aerobic display for some colour variables suggests that comparisons between production systems at one time point should be made with caution. Holman et al. [9] concluded that a* provides the most simple and robust prediction of consumer acceptability of beef colour. When a* was equal to, or above, 14.5, samples were acceptable to the consumer with a 95% confidence interval. Based on this criterion, beef from the four production systems examined would be acceptable after 24 h of aerobic display. 

Mezgebo et al. [10] observed no difference in colour of LT from EM and LM breed sired suckler bulls, finished on concentrates after 100 days at pasture. Similarly, there was no difference in lightness, redness and yellowness of LT from Charolais (or Limousin (LM breed)) or Angus (EM breed) sired bulls fed a high-concentrate ration [30]. In contrast, Cuvelier et al. [31] found LT from Limousin sired bulls to be lighter than LT from Angus sired bulls, redness did not differ and other colour variables were not reported. In a survey to 15 European breeds, higher lightness and lower redness in LT from Charolais (and Limousin) compared to Angus sired bulls were observed [32].

### 4.3. Intramuscular Fat Concentration

The lower IMF concentration in LT from the LM breed sired bulls observed in the present study was as expected [30]. The IMF concentration in LT from all groups in the present study is lower than reported in the studies cited above (IMF for the bulls in [32] is given in [33]. Consequently, grass-fed bull beef may be attractive to European consumers in particular, who prefer beef with less visual fat [34]. 

### 4.4. Eating Quality

Historically, tenderness was considered to be the most important driver of satisfaction when beef was being consumed and this led to a focus on understanding and controlling factors that influence tenderness [35] and the development of objective measurements of tenderness such as WBSF. The moderate negative association between the two measures of tenderness in the present study is similar to that previously reported [5,12] suggesting that WBSF may not always be a reliable indicator of tenderness as perceived by the consumer. Recent consumer studies in Europe [36] and the United States [35] indicate that consumers now consider flavour to be as, or slightly more, important as tenderness, which they conclude reflects improvements in the management of tenderness over time. Since bulls are usually produced indoors on rations with a high energy concentration, there is a paucity of data on the eating quality characteristics of beef from bulls slaughtered from pasture and in particular from pasture with a high nutritive value. With respect to breed maturity effects on intramuscular connective tissue characteristics, LT from Angus-sired bulls tends to have a higher concentration of collagen and a higher proportion of soluble collagen than Charolais-sired suckler bulls, albeit the differences are not always statistically significant [10,33,37], consistent with findings in the present study. The structure of collagen was examined by measuring the concentration of proteoglycans which are involved in stabilising the collagen network in muscle [38] and thermo-stable cross-links (pyridinoline and Ehrich’s chromogen) considered to be a major influence on residual toughness after ageing and cooking [16]. The higher perimysium collagen and pyridinoline concentrations in muscle and from the EM breed sired bulls in the present study was previously observed in a comparison of EM and LM breed sired steers [16], while higher pyridinoline was also reported for purebred Angus compared to Limousin bulls [38]. Roy et al. [16] suggested that this was related to the greater physiological maturity of EM breeds. Despite the difference in collagen structure, the sensory tenderness or WBSF of LT from the EM breed sired bulls was similar to that of the LM breed sired bulls. Nishimura [39] has outlined how IMF deposition disrupts the structure of the perimysium and contributes to an increase in tenderness. The 3-fold higher IMF concentration in LT from EM breed sired bulls appears to have been sufficient to disrupt the increase in collagen crosslinks and prevent a decrease in tenderness. When WBSF of LT from EM (Angus) and LM breed (Charolais and/or Limousin) sired bulls was compared, no difference was observed [31,32,40]. For comparisons of sensory characteristics between EM and LM bulls, there was no difference in tenderness [40,41] or tenderness was rated higher for EM breed bulls [10,42], there was no difference in flavour [10,40] or flavour was rated higher for EM breed bulls [41,42] and there was no difference in juiciness [10,40,41]. Within a database of 1318 bulls of 8 breeds, Judge et al. [23] reported sensory tenderness ratings of 7.07, 6.94 and 6.98 (scale 1-10) for Angus, Charolais and Limousin breeds, respectively. Corresponding ratings for flavour were 6.72, 6.52 and 6.55, and for juiciness were 6.62, 6.42 and 6.43. These data support those summarised above, i.e., differences in sensory characteristics are generally small between EM and LM breed bulls and in particular when IMF concentration is similar. Indeed, after a more in-depth analysis of the data for 15 breeds reported in [41], Conanec et al. [43] concluded that “differences in sensory scores between most of the breeds were small with only significant differences between the few breeds that had extreme sensory profiles such as Simmental and Pirenaica”.

There are few grazing studies in the literature that can be directly compared with the present study, e.g., in some studies animals assigned to a stated “unsupplemented grazing” treatment required some supplementation to compensate for poor pasture quality during the course of the study and so did not differ substantially from the supplemented group [44]. When Eastern Anatolian Red bulls grazed a mixed pasture (35% crested wheatgrass, 30% white clover, 20% orchard grass and 15% perennial ryegrass) alone or supplemented with concentrates at 1.5% bodyweight for 93 days, there was no difference in tenderness (or WBSF), juiciness, flavour intensity or overall acceptability of beef [45]. This is consistent with the findings of Moloney et al. [6] who compared the eating quality of LT from LM bulls finished at pasture without or with concentrate supplementation or indoors on concentrates, but not with the present study where concentrate supplementation decreased sensory tenderness and increased WBSF. This appears to be related to a greater perimysium collagen fraction and associated collagen cross–links in the concentrate-supplemented bulls, i.e., they have attained a greater proportion of their mature weight at slaughter compared to unsupplemented bulls. As discussed above, IMF deposition disrupts the structure of the perimysium and contributes to an increase in tenderness [39]. It is possible, therefore, that the small increase in IMF concentration due to concentrate supplementation (1.3-fold) was not sufficient to disrupt the increase in collagen crosslinks due to greater maturity, leading to a decrease in tenderness. The discrepancy between studies likely reflects the difference in magnitude of the carcass growth response to concentrate supplementation, 28 kg on average in the present study and 13 kg in [6], i.e., concentrate supplementation in the present study having a greater effect on physiological maturity. With respect to the absolute difference in tenderness, it did not influence overall acceptability and so, of itself, is unimportant. In support of this, Conroy et al. [46] reported that Irish consumers across a range of age categories did not consistently distinguish the acceptability of beef that had WBSF values that spanned 15N, compared to the increase in 3.6N due to concentrate supplementation on the present study.

## 5. Conclusions

The data from the present study confirm that carcass fat score is a poor indicator of the eating quality of grass-fed suckler bull beef. Consequently, suckler bulls can be produced at grass to achieve an acceptable sensory quality which is not influenced by breed type or supplementary concentrate feeding. However, while the current carcass specification remains, an EM sire breed reduces the risk of non-compliance with the carcass fatness requirement while meeting the definition of grass-fed and also being eligible for the current premium breed market. The basis for this premium, based on the current study and the literature cited, is rather weak for bull beef.

## Figures and Tables

**Table 1:** Colour of longissimus muscle from early or late maturing suckler bulls, finished at pasture with or without concentrate supplementation.

**Breed Type (BT)**	**Late Maturing**		**Early Maturing**		**Mean**
**Concentrates (C, g/kg)**	**0**	**400**	**0**	**400**	
Lightness ^1^					
1 h ^2^	41.9	41.8	41.9	41.2	41.70
24 h	44.1	43.9	43.8	43.4	43.80
Mean	43.0	42.9	42.8	42.3	
**s.e.d.**	0.64				
**Significance ^3^**					
BT	NS				
C	NS				
BT*C	NS				
Time	***				
Time*BT	NS				
Time*C	NS				
Time*BT*C	NS				
Redness ^1^					
1 h	12.9	12.3	13.0	12.7	12.7
24 h	15.4	15.3	14.8	15.0	15.1
Mean	14.1	13.8	13.9	13.9	
**s.e.d.**	0.47				
**Significance**					
BT	NS				
C	NS				
BT*C	NS				
Time	***				
Time*BT	0.078				
Time*C	NS				
Time*BT*C	NS				
Yellowness ^1^					
1 h	9.4	8.8	9.4	8.9	9.1
24 h	12.5	12.2	12.2	12.2	12.3
Mean	11.0	10.5	10.8	10.6	
**s.e.d.**	0.36				
**Significance**					
BT	NS				
C	NS				
BT*C	NS				
Time	***				
Time*BT	NS				
Time*C	NS				
Time*BT*C	NS				
**Breed Type (BT)**	**Late Maturing**		**Early Maturing**		**Mean**
**Concentrates (C, g/kg)**	**0**	**400**	**0**	**400**	
Chroma ^1^					
1 h	16.0	15.1	16.0	15.5	15.7
24 h	19.9	19.6	19.2	19.4	19.5
Mean	17.9	17.3	17.6	17.5	
**s.e.d.**	0.55				
**Significance**					
BT	NS				
C	NS				
BT*C	NS				
Time	***				
Time*BT	NS				
Time*C	NS				
Time*BT*C	NS				
Hue ^1^					
1 h	36.0	35.6	35.8	35.0	35.6
24 h	39.4	38.6	39.5	39.2	39.2
Mean	37.7	37.1	37.7	37.1	
**s.e.d.**	0.68				
**Significance**					
BT	NS				
C	NS				
BT*C	NS				
Time	***				
Time*BT	0.056				
Time*C	NS				
Time*BT*C	NS				

^1^ L*, a*, b* = lightness, redness and yellowness, respectively, For colour variables, higher values indicate lighter, more red, more yellow, more saturated (chroma) and higher hue. ^2^ After 1 or 24 h aerobic exposure. ^3^ NS = not significant, *** = *p* <0.001, S.e.d. = standard error of the difference between means.

**Table 2:** pH and composition of longissimus muscle from early or late maturing suckler bulls finished at pasture with or without concentrate supplementation.

Breed Type (BT)	Late Maturing		Early Maturing		S.e.d.	Significance ^1^		
Concentrates (C, g/kg)	0	400	0	400		BT	C	BT*C
pH	5.52	5.51	5.50	5.51	0.023	NS	NS	NS
Composition (g/kg muscle)								
Lipid	1.8	2.1	4.7	6.4	1.86	**	NS	NS
Protein	229.3	229.4	230.6	228.2	16.2	NS	NS	NS
Moisture	752.5	749.3	749.6	753.6	3.7	NS	NS	NS
Soluble collagen (g/kg muscle)	0.50	0.48	0.56	0.56	0.058	NS	NS	NS
Insoluble collagen (g/kg muscle)	4.61	4.37	4.57	4.85	0.439	NS	NS	NS
Total collagen (g/kg muscle)	5.11	4.85	5.13	5.40	0.481	NS	NS	NS
Collagen solubility (g/kg)	97.3	97.9	109.9	105.3	7.03	*	NS	NS
Proteoglycans (mg/kg muscle)	12.47	13.38	13.2	14.75	1.688	NS	NS	NS
Proteoglycans (g/kg collagen)	2.54	2.78	2.65	2.93	0.387	NS	NS	NS
Perimysial collagen (PC) (g/kg muscle)	3.01	3.44	3.52	4.16	0.226	***	**	NS
Pyridinoline (mol/mol PC collagen)	0.36	0.34	0.33	0.35	0.019	NS	NS	NS
Pyridinoline (mmol/kg muscle)	3.58	3.96	3.85	4.86	0.340	*	**	NS
Ehrlich chromogen (mol/mol PC collagen)	0.39	0.37	0.37	0.39	0.013	NS	NS	NS
Ehrlich chromogen (mmol/kg muscle)	3.90	4.30	4.31	5.39	0.348	**	**	NS

^1^ NS = not significant, * = *p* <0.05, ** = *p* < 0.01, *** = *p* <0.001, S.e.d. = standard error of the difference between means.

**Table 3:** Warner–Bratzler shear force (WBSF), cook loss and sensory characteristics of longissimus muscle from early or late maturing suckler bulls finished at pasture with or without concentrate supplementation.

Breed Type (BT)	Late Maturing		Early Maturing		S.e.d.	Significance ^1^		
Concentrates (C, g/kg)	0	400	0	400		BT	C	BT*C
WBSF (N)	37.4	43.3	37.1	38.4	2.46	NS	*	NS
Cook loss (g/kg)	292.6	296.1	293.4	303.2	8.58	NS	NS	NS
Sensory characteristics ^2^								
Tenderness	4.07	3.74	4.48	3.92	0.218	0.058	**	NS
Flavour	4.10	4.03	4.32	4.09	0.187	NS	NS	NS
Firmness	5.12	5.38	5.02	5.40	0.229	NS	0.053	NS
Texture	3.78	3.73	4.27	3.85	0.229	0.067	NS	NS
Acceptability	3.95	3.78	4.34	3.99	0.234	0.080	NS	NS

^1^ NS = not significant, * = *p* <0.05, ** = *p* <0.01, S.e.d. = standard error of the difference between means ^2^ Scale: tenderness (1=extremely tough, 8=extremely tender), flavour (1 = very poor, 8 = very good), firmness (1 = very mushy, 8 = very firm), texture (1 = very poor, 8 = very good), overall acceptability (1 = not acceptable, 8 = extremely acceptable).

**Table 4:** Correlation between intramuscular fat concentration, collagen characteristics and sensory characteristics of longissimus.muscle after adjustment for treatment effects.

	WBSF	Tenderness	Flavour	Firmness	Texture	Acceptability
Fat score	0.01	−0.01	−0.01	0.09	0.05	−0.04
IMF (g/kg muscle)	−0.17	0.05	0.06	−0.15	0.09	0.09
Collagen (g/kg muscle)	−0.21	−0.08	−0.03	0.04	−0.05	0.02
Collagen solubility (g/kg)	0.16	0.10	−0.01	0.04	0.11	0.02
Proteoglycans (mg/kg muscle)	0.05	−0.13	−0.10	0.10	0.02	−0.07
Proteoglycans (g/kg collagen)	0.15	−0.04	−0.07	0.07	0.11	0.03
Perimysium collagen (g/kg muscle)	0.08	−0.12	−0.01	0.01	−0.04	−0.04
Pyridinoline (mol/mol perimysium collagen)	−0.08	0.17	−0.01	−0.01	0.02	0.02
Pyridinoline (mmol/kg muscle)	0.01	−0.03	−0.08	0.09	−0.07	−0.09
Ehrlich chromogen (mol/mol perimysium collagen)	−0.06	−0.15	−0.09	0.09	−0.16	−0.06
Ehrlich chromogen (mmol/kg muscle)	−0.01	−0.19	−0.10	0.12	−0.11	−0.11

## Data Availability

The data presented in this study are available on request from the corresponding author.
